# Estimates of missing heritability for complex traits in Brown Swiss cattle

**DOI:** 10.1186/1297-9686-46-36

**Published:** 2014-06-04

**Authors:** Sergio-Iván Román-Ponce, Antonia B Samoré, Marlies A Dolezal, Alessandro Bagnato, Theo HE Meuwissen

**Affiliations:** 1 Dipartimento di Scienze e Tecnologie Veterinarie per la Sicurezza Alimentare, Università degli Studi di Milano, Via Celoria 10, Milano 20133, Italia; 2 Department of Animal and Aquacultural Sciences, Norwegian University of Life Sciences, P.O. Box 5003, Oslo N-1432 Ås, Norway; 3 Instituto Nacional de Investigaciones Forestales Agrícolas y Pecuarias, C.E. Valles Centrales, CIRPAS, Melchor Ocampo 7, Etla, Oaxaca 68200, México

## Abstract

**Background:**

Genomic selection estimates genetic merit based on dense SNP (single nucleotide polymorphism) genotypes and phenotypes. This requires that SNPs explain a large fraction of the genetic variance. The objectives of this work were: (1) to estimate the fraction of genetic variance explained by dense genome-wide markers using 54 K SNP chip genotyping, and (2) to evaluate the effect of alternative marker-based relationship matrices and corrections for the base population on the fraction of the genetic variance explained by markers.

**Methods:**

Two alternative marker-based relationship matrices were estimated using 35 706 SNPs on 1086 dairy bulls. Both pedigree- and marker-based relationship matrices were fitted simultaneously or separately in an animal model to estimate the fraction of variance not explained by the markers, i.e. the fraction explained by the pedigree. The phenotypes considered in the analysis were the deregressed estimated breeding values (dEBV) for milk, fat and protein yield and for somatic cell score (SCS).

**Results:**

When dEBV were not sufficiently accurate (50 or 70%), the estimated fraction of the genetic variance explained by the markers was around 65% for yield traits and 45% for SCS. Scaling marker genotypes with locus-specific frequencies of heterozygotes slightly increased the variance explained by markers, compared with scaling with the average frequency of heterozygotes across loci. The estimated fraction of the genetic variance explained by the markers using separately both relationships matrices followed the same trends but the results were underestimated. With less accurate dEBV estimates, the fraction of the genetic variance explained by markers was underestimated, which is probably an artifact due to the dEBV being estimated by a pedigree-based animal model.

**Conclusions:**

When using only highly accurate dEBV, the proportion of the genetic variance explained by the Illumina 54 K SNP chip was approximately 80% for Brown Swiss cattle. These results depend on the SNP chip used and the family structure of the population, i.e. more dense SNPs and closer family relationships are expected to result in a higher fraction of the variance explained by the SNPs.

## Background

Genome-wide dense marker arrays that are available for livestock populations cover all chromosomes with dense single nucleotide polymorphism (SNP) markers [1]. Many dairy cattle populations are currently being genotyped using these arrays [2-4]. The main objective is to apply genomic selection (GS) [5]. GS allows prediction of the genetic merit of young animals based on marker information in the absence of own performance data. The marker effects are estimated in a reference population, which must have both genotypic and phenotypic records. In the case of dairy bulls, phenotypic data come from genetic evaluations in the form of daughter yield deviation (DYD) or deregressed estimated breeding values (dEBV) [6].

Identity by descent (IBD) alleles refer to alleles that descend from a common ancestor in the base population [7]. The coefficient of coancestry between two animals is defined as the probability that two randomly sampled alleles from the two animals are IBD [8], and twice the coancestry is defined as their numerator relationship [8]. This approach leads to the estimation of a matrix of relationships based on the pedigree information. The latter is fundamental to estimate the genetic parameters for complex traits such as heritability (defined as the proportion of the phenotypic variance in a population that is attributed to additive genetic effects). The relationship matrix based on pedigree data dates back to a base population, for which parents are unknown and which is considered unrelated, unselected and non-inbred. The choice of the base population affects the estimate of the additive genetic variance [9].

However, the relationship matrix can also be estimated from genome-wide genetic markers such as panels of SNPs [10-12]. Methods have been developed to construct such marker-based relationship matrices [12-15]. Recently, these relationship matrices have been used to dissect the additive genetic variance of complex traits [16].

The proportion of the genetic variance not captured by markers (*C*_*miss*_) represents the variance that cannot be used by GS and affects the maximum accuracy that can be achieved by GS [17]. The term ‘missing heritability’ [18] describes the fact that marker-phenotype associations identified in genome-wide association studies do not explain all the genetic variance in complex traits (e.g. height in humans). Some strategies have been proposed to reduce *C*_*miss*_: (1) increasing the sample size in order to also detect genes with smaller effects, (2) expanding the studies to non-European samples in human genetics, (3) enlarging the collection of phenotypes to explore gene-gene interactions, (4) changing the structure of the training population, mainly in terms of the relatedness of the included individuals, and (5) moving to the genomic selection approach instead of estimating the marker effect for each SNP individually [13,19,20]. In animal breeding, some results suggest that the Illumina Bovine54K chip array (Illumina Inc., San Diego, CA) does not capture all the additive genetic variation for all dairy traits [21-23], even when using the GS approach, it estimates simultaneously all the SNP effects.

The main objective of this study was to estimate the fraction of the genetic variance not explained by the 54 K Illumina SNP chip. Two alternative marker-based relationship matrices were used for analysis.

## Methods

### Genotypic and phenotypic data

**Figure 1:**
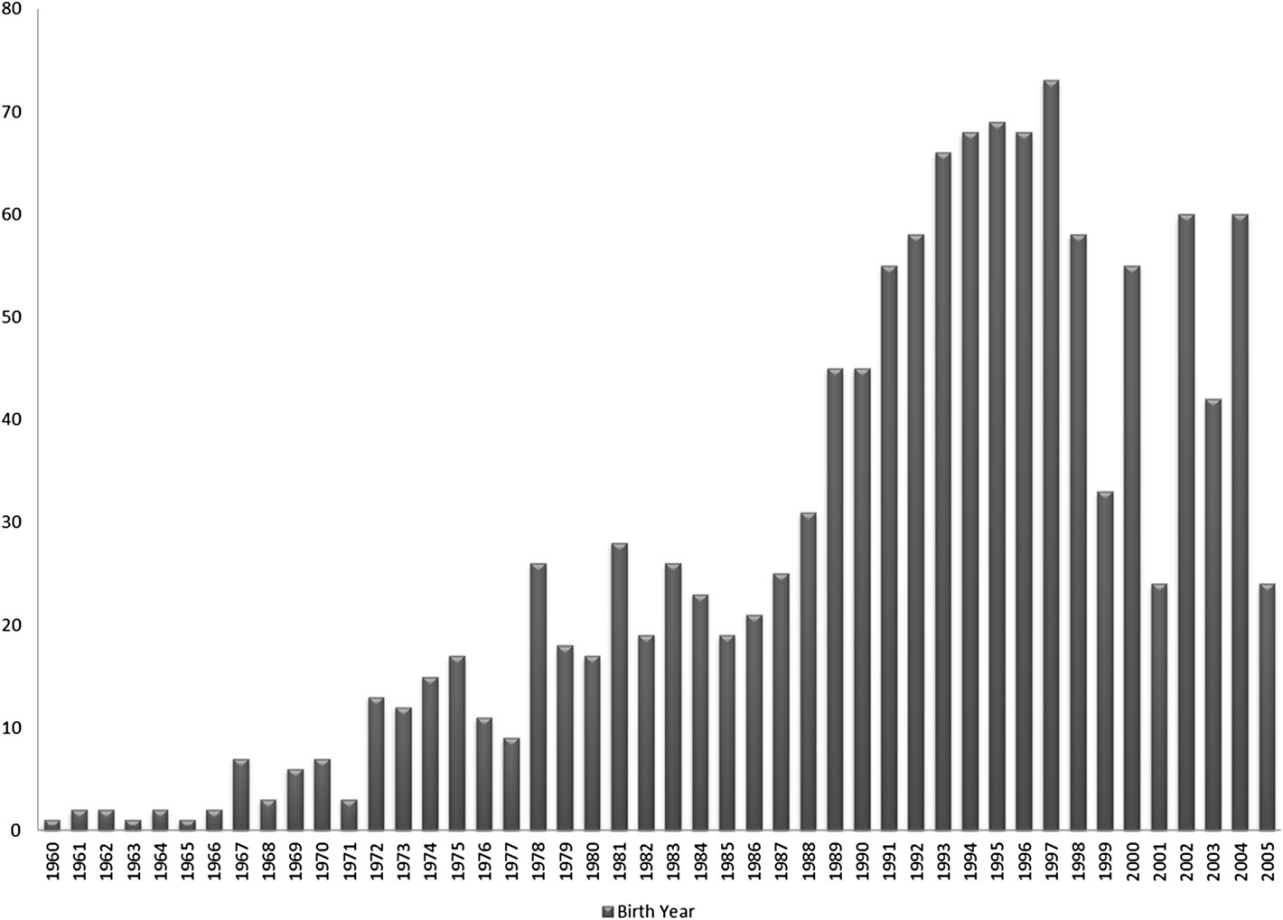
Distribution of birth years of the 1092 genotypes Italian Brown Swiss bulls.

A total of 1092 Italian Brown Swiss bulls were genotyped with the Illumina Bovine54K chip (Illumina Inc., San Diego, CA). These bulls were born between 1963 and 2002. Figure 1 shows the distribution of the genotyped bulls over the birth years. All the SNPs on the X-chromosome were excluded from the analysis, which left 51 582 markers. The quality control process removed 1421 SNPs that had more than 5% missing genotypes and 14 455 SNPs with a minor allele frequency lower than 5%. Six sires were deleted because their genotyping rate was lower than 95%. Editing was performed with two different software packages: SAS^®^ (SAS Inst. Inc., Cary, NC) and PLINK v1.07 [24]. At the end of the quality control process, genotypes were available for 1086 sires with 35 706 SNPs and with a missing genotype rate of 0.66%.

The phenotypic data available were the EBV for fat yield (FAT), milk yield (MILK), protein yield (PROT) and somatic cell score in milk (SCS) for each bull, which were calculated by the Italian National Association of Brown Swiss (ANARB). The EBV were deregressed as proposed by Garrick [21], in order to eliminate the shrinkage contained in the EBV and to remove ancestral information. The deregressed EBV (dEBV) were used as phenotypic records for the bulls with heritability equal to the reliability of the EBV.

Three subsets were formed according to the reliability of EBV as follows: animals with a reliability of at least 50% for each trait; animals with a reliability greater than 70% for each trait; animals with a reliability of at least 90% for each trait.

### Relationship matrices: A and G

A pedigree file was extracted from the Italian Brown Swiss herd book. Pedigree was traced back five generations and the pedigree file included 6826 entries. The completeness in the pedigree was 100% up to the grandparents, and decreased to ~90% thereafter. The equivalent number of known generations as calculated by the software Pedig [25] was on average 5.14 and the median was 5.23. The pedigree file was used to estimate the additive genetic relationships (**A**) with an adapted version of the procedure proposed by Meuwissen and Luo [26], as implemented in ASREML [27].

Two genomic relationship matrices (**G**) were computed for all genotyped animals. The first **G**_**V**_ was based on the method proposed by VanRaden [12]. Let **M** be the marker-genotype matrix with number of individuals (n) and number of loci (m) as dimensions. The elements in the matrix **M** were coded as -1 (homozygous for one allele) 0 (heterozygous) and 1 for (homozygous for the other allele). The nxm matrix **P** contains columns with all elements 2(*p*_i_-0.5), where *p*_i_ is the frequency of the second allele at locus *i*. The matrix **P** was subtracted from **M** to give **Z = M - P**. Finally, matrix **G**_**V**_ was calculated as:

$$G_{V}=\frac{ZZ^{'}}{2\sum_{i=1}^{m}p_{i}\left(1-p_{i}\right)}.$$

The second genomic relationship matrix (**G**_**Y**_) was computed as:

$$\;G_{Y}=\frac{WW^{'}}{m},$$

where **W** is the **Z** matrix but with each element scaled based on the allele frequency of each locus as follows: $w_{\mathrm{ij}}=\frac{Z_{\mathrm{ij}}}{\sqrt{2p_{j}\left(1-p_{j}\right)}}$[12,14].

### Correction for the base population

Both the **G** matrix and the pedigree-based relationship matrix, **A**, are expressed relative to a base population, i.e. an original population in which all animals are assumed unrelated and non-inbred, and these populations may differ between the pedigree-based and genomic relationship matrices [15]. To correct for these differences, the scale of **G** was changed to that of **A** based on Wright’s F-statistic [7]. We expressed the total inbreeding of animal *i* in the **G** matrix as:

$$F_{\mathrm{it}}=G_{\mathrm{ii}}-1\;\mathrm{or}\;F_{\mathrm{it}}=F_{\mathrm{st}}+\left(1-F_{\mathrm{st}}\right)\;F_{\mathrm{is}},$$

where *F*_*st*_ is the average inbreeding in the population, i.e. the average of the diagonal elements of **G** minus 1, and *F*_*is*_ is the inbreeding of animal *i* relative to the population average inbreeding *F*_*st*_, which is calculated as: $F_{\mathrm{is}}=\frac{\left(F_{\mathrm{it}}-F_{\mathrm{st}}\right)}{\left(1-F_{\mathrm{st}}\right)}=\frac{\left(G_{\mathrm{ii}}-1-F_{\mathrm{st}}\right)}{\left(1-F_{\mathrm{st}}\right)}.$

The average population inbreeding of **G** was set equal to that of **A** by rescaling the diagonal element of **G** corresponding to individual *i* as:

$$G_{\mathrm{jj}}^{*}=A_{\mathrm{st}}+\left(1-A_{\mathrm{st}}\right)F_{\mathrm{st}}+1,$$

Where *A*_*st*_ is the average of the diagonals of **A** minus 1. The off-diagonals of **G** were rescaled similarly, using the same *F*_*st*_ and *A*_*st*_ values. Numerator relationships were transformed to kinships, ∅, i.e. by dividing the relationship by 2, and performing the base-correction on the kinship level, which is the same level as that of inbreeding, i.e.

$$\emptyset_{\text{jis}}=\frac{\left(\frac{G_{\mathrm{ji}}}{2}-F_{\mathrm{st}}\right)}{\left(1-F_{\mathrm{st}}\right)},\text{and}$$

$$G_{\mathrm{ji}}^{*}=2\left[A_{\mathrm{st}}+\left(1-A_{\mathrm{st}}\right)\emptyset_{\text{jis}}\right],$$

where ∅ _*jis*_ is the kinship of animal *j* and *i* relative to the base population inbreeding, *F*_*st*_.

### Estimation of variance components

To estimate the fraction of the genetic variance captured by dense markers covering the entire genome, the approach of Goddard et al. [28] was used. Both matrix **A** and **G** were fitted in the model simultaneously in order to estimate the fraction of the genetic variance captured by each of these matrices. The variance component analyses were performed by ASREML-R [29], using the following model:

$$\;y=1\mu +Z_{1}a+Z_{2}u+e,$$

where **y** is the vector of the dEBV; μ is the overall mean; **Z**_**1**_ and **Z**_**2**_ are the incidence matrices for pedigree-based and genomic random animal effects, respectively; **a** is the vector of the random additive genetic animal effects using the pedigree-based relationship matrix, with **a** ~ N(0, **A**σ^2^_**a**_); **u** is the vector of random additive genetic effect using the genomic relationship matrix, with **u** ~ N(0, **G**σ^2^_**u**_); and finally, **e** is the vector of random residual effects. Because the number of daughters per bull was high for all bulls, the reliabilities of the dEBV were high and varied little between bulls, and a homogeneous error variance structure was assumed.

If we assume that **A** is an unbiased estimate of **G**, and write **G** = **A** + **D**[28], where **D** is a matrix of deviations from pedigree relationships due to the segregation of a finite number of chromosome segments in the genome, the genetic variance of the records becomes *V*(**g**) = **G**σ_*u*_^2^ + **A**σ_*a*_^2^ = **A**(σ_*u*_^2^ + σ_*a*_^2^) + **D**σ_*u*_^2^. Hence, as in a model that fits only pedigree relationships (**y** = **1**μ + **Z**_1_**a** + **e**), the total genetic variance is explained by the **A** matrix and the segregation of chromosome segments that are traced by the markers is explained by σ_*u*_^2^. The fraction of genetic variance not captured by the markers on the SNP chip (*C*_*miss*_) was thus estimated as:

$$C_{\text{miss}}=1-\frac{\sigma_{u}^{2}}{\sigma_{g}^{2}}=1-\frac{\sigma_{u}^{2}}{\left(\sigma_{a}^{2}+\sigma_{u}^{2}\right)},$$

where *σ*^2^_*g*_ is the total genetic variance, *σ*^2^_*u*_ is the variance due to marker-based relationships and *σ*^2^_*a*_ is the variance due to pedigree-based relationships.

The two additive genetic variances were also estimated by fitting each separately: the additive genetic animal variance using the pedigree-based relationship matrix ($\sigma_{a0}^{2}$) and the additive genetic variance using the genomic relationship matrix ($\sigma_{g0}^{2}$). The estimate of $\sigma_{a0}^{2}$ was used to calculate an alternative estimate for the fraction of genetic variance not addressed by the markers on the SNP chip (*C*_*miss*2_) as follows: $C_{\text{miss}2}\;=\;1\;-\;\frac{\sigma_{u0}^{2}}{\sigma_{a0}^{2}}$. The estimate *C*_*miss*2_  has the advantage that *σ*^2^_*a*0_ is known to yield an unbiased estimate of the genetic variance, but it has the disadvantage that *σ*^2^_*u*0_ is likely to include more genetic variance than that explained by QTL that are in LD with the markers [11]. E.g. if only some of the chromosomes contain markers, these markers can explain genetic variance at the unmarked chromosomes, because the markers trace family relationships. If, in the latter case, the pedigree-based relationship matrix is fitted simultaneously with the marker-based relationship matrix, the variance due to the unmarked chromosomes is expected to be included in the polygenic variance, *σ*^2^_*a*_, because the pedigree-based relationship matrix more closely resembles the family relationships at the unmarked chromosomes than at the marked chromosomes, which may show relationships that (randomly) deviate from the pedigree. Thus, *C*_*miss*2_ is expected to underestimate the fraction of missing genetic variance.

## Results

### Descriptive statistics

Descriptive statistics for each trait and dataset are in Table 1. In the group of bulls with dEBV reliabilities of at least 50%, the dEBV average reliability was ~90% (±7%) for the production traits (FAT, PROT and MILK), and 82.6% (±10.7%) for SCS. The subset of sires with dEBV reliabilities of at least 70% had a similar average reliability of ~91% (±5%) for the production traits. The lowest average reliability in this subset was 85.7% (±7.4%) for SCS. Finally, the subset of bulls with reliabilities of at least 90% had an average reliability close to ~94% (±3%) for all traits. As expected, the differences in the average of the reliabilities between traits tended to decrease with increasing minimum reliability requirements.

**Table 1:** **Descriptive statistics for de-regressed estimated breeding values (dEBV) and reliabilities (r**^**2**^) for production traits*

**Trait**	**Subset label**	**Number of observations**	**dEBV**		**r**^**2 **^**(%)**	
			**Mean**	**SD**	**Mean**	**SD**
Fat yield	dFAT50	1034	-8.1	26.3	90.2	7.4
	dFAT70	1006	-8.7	26.1	91.0	5.8
	dFAT90	655	-12.7	25.4	94.3	2.9
Milk yield	dMILK50	1034	-205.9	666.9	90.7	7.3
	dMILK70	1014	-214.7	665.6	91.4	5.6
	dMILK90	691	-316.1	646.9	94.4	2.9
Protein yield	dPROT50	1034	-8.2	23.3	90.6	7.1
	dPROT70	1009	-8.7	23.2	91.3	5.6
	dPROT90	681	-12.1	22.9	94.4	2.9
Somatic cell score	dSCS50	978	0.246	1.206	82.6	10.7
	dSCS70	848	0.233	1.118	85.7	7.4
	dSCS90	223	0.018	0.972	95.2	2.9

*Subsets of the genotyped sire population were divided based on minimum reliabilities (50, 70, or 90); SD: standard deviation.

### Proportion of genetic variance not explained by markers

The fraction of genetic variance not explained by molecular markers based on *C*_*miss*_ was estimated for all datasets (50, 70 and 90 dEBV reliabilities) and traits (FAT, PROT, MILK and SCS). Results are in Table 2. For dFAT50, the estimate of *C*_*miss*_ was 0.373 ± 0.068 based on **G**_**V**_ and 0.363 ± 0.069 based on **G**_**Y**_. The estimates of *C*_*miss*_ were smaller for the dFAT70 subset than for the dFAT50 subset. For dFAT90, the estimate was 0.305 ± 0.074 **G**_**V**_, while the **G**_**Y**_ matrix did not result in converged variance component estimates. Algorithms other than the AI-REML algorithm might have converged (e.g. the EM-algorithm, which is known to be slow), but the convergence difficulties are probably due to the small size of the dataset, thus resulting variance component estimates would have been unreliable.

**Table 2:** **Proportion of genetic variance not explained by markers (***C*_*miss*_) ± standard error (SE) for dEBV for production traits*^1^

**Label**	**G**_**Y**_	**G**_**V**_
dFAT50	0.363 ± 0.069	0.373 ± 0.068
dFAT70	0.363 ± 0.072	0.369 ± 0.070
dFAT90	NC	0.305 ± 0.074
dMILK50	0.337 ± 0.076	0.357 ± 0.074
dMILK70	0.342 ± 0.077	0.358 ± 0.075
dMILK90	0.199 ± 0.101	0.245 ± 0.098
dPROT50	0.345 ± 0.077	0.363 ± 0.074
dPROT70	0.344 ± 0.078	0.357 ± 0.076
dPROT90	0.206 ± 0.098	0.235 ± 0.095
dSCS50	0.486 ± 0.095	0.532 ± 0.091
dSCS70	0.492 ± 0.101	0.530 ± 0.097
dSCS90	0.061 ± 0.197	NC

*Subsets of the genotyped sire population were divided based on minimum reliabilities (50, 70, or 90); NC: Log-likelihood was not available since the iterative procedure was not convergent; ^1^**G**_**Y**_, and **G**_**V**_: genomic relationship matrices as proposed by [14] and [12], respectively, and corrected to the same base population.

The fraction of the genetic variance not explained by molecular markers based on *C*_*miss*2_ through the additive genetic variances was estimated separately for all datasets and traits (Table 3). Results for *C*_*miss*2_ followed the same trends as for *C*_*miss*_ but the values of *C*_*miss*2_ were lower probably due to its underestimation of the fraction of the missing genetic variance.

**Table 3:** **Proportion of genetic variance not explained by markers (***C*_*miss*2_) for dEBV for production traits*^1^

**Label**	**G**_**Y**_	**G**_**V**_
dFAT50	0.116	0.097
dFAT70	0.108	0.089
dFAT90	0.026	0.024
dMILK50	0.073	0.055
dMILK70	0.075	0.057
dMILK90	0.125	0.101
dPROT50	0.054	0.035
dPROT70	0.052	0.031
dPROT90	0.031	0.008
dSCS50	0.149	0.149
dSCS70	0.152	0.152
dSCS90	-0.024	-0.024

*Subsets of the genotyped sire population were divided based on minimum reliabilities (50, 70, or 90); ^1^**G**_**Y**_ and **G**_**V**_: genomic relationship matrices as proposed by [14] and [12], respectively.

Results for dMILK, dPROT and dSCS were similar to those described above for dFAT for both genomic relationship matrices. Estimates of *C*_*miss*_ for dMILK70 and dPROT70 hardly differed from those for dMILK50 and dPROT50, respectively. The subsets with dEBV90 resulted in estimates of *C*_*miss*_ of 0.199 (±0.101) for dMILK90 and 0.206 (±0.098) for dPROT90 when using **G**_**Y**_. These estimates were not significantly different from those obtained with the larger datasets for the same traits (dEBV50 or dEBV70), although they were systematically lower for all traits.

The highest estimates for *C*_*miss*_ were obtained for dSCS50, with 0.532 (±0.091) for **G**_**V**_. When using **G**_**Y**_, the corresponding *C*_*miss*_ estimate was lower (0.486 ± 0.095). The smallest *C*_*miss*_ estimate was obtained for dSCS90: 0.061 (±0.197) using **G**_**Y**_. The variance component analysis with **G**_**V**_ on the same dataset did not converge. This was the smallest dataset and, although the average reliability was the highest, estimates of *C*_*miss*_ were not significantly different from 0.

In general, estimates of *C*_*miss*2_ decreased as the reliability of the dEBV increased. Estimates of *C*_*miss*2_ differed from estimates of *C*_*miss*_, probably because *C*_*miss2*_ is expected to underestimate the fraction of the missing genetic variance.

## Discussion

We estimated the fraction of the genetic variance not accounted by SNPs in the marker panel (*C*_*miss*_) based on the Illumina 54 K SNP chip for complex traits in dairy cattle. The results showed that the estimates of *C*_*miss*_ depended on the reliability of the phenotypic traits considered, i.e. the dEBV used as response values. When the accuracy of the dEBV increases, i.e. when the correlation between dEBV and the true breeding value increases, the proportion of the genetic variance explained by SNPs tended to increase. When the reliability of the dEBV is low, the family/pedigree information greatly contributes to the estimation of the EBV, which results in a larger fraction of the variance being explained by **A** and, in turn, in upward biases of *C*_*miss*_. Because the estimates of the *C*_*miss*_ values, are expected to be overestimated due to the use of (family information in) dEBV, the best estimates of *C*_*miss*_ are obtained for data sets with high reliabilities, which resulted in estimates around 0.2. This implies that the maximum accuracy of GEBV is √(1-*C*_*miss*_) ≈ 0.9, which agrees with the result of Daetwyler [22], who studied the increase in the accuracy of GEBV with increasing training population sizes.

For all production traits, the fraction of the genetic variance not explained by the SNPs was significantly different from 0, even when the phenotypes were very accurate (reliability > 90%), and were, therefore, very close to the true breeding values. Correction for the base population did not affect the fraction of the genetic variance explained by markers for any of the marker-based relationships here used. The differences in *C*_**miss**_ estimates between using **G**_**V**_ and **G**_**Y**_ were negligible for all traits and all subsets. Similarly, when using EBV instead of dEBV (results not shown), the results were virtually the same.

If original performance records of production and SCS phenotypes are used to estimates *C*_*miss*_, instead of dEBV, the upward biases mentioned above are not expected to occur. The error variances would be higher than when using dEBV, but the value of σ^2^_a_ would not be inflated, because family information does not contribute to own phenotype (in contrast to dEBV phenotypes).

The sources of phenotypic information used in genomic analyses are very heterogeneous and vary from individuals with highly reliable information, i.e. progeny-tested bulls, and animals with phenotypes with low levels of accuracy, i.e. young cows. To take into account these differences in reliability in a weighted analysis, it is necessary to know the value of *C*_*miss*_ for each phenotype [22]. In addition, a polygenic effect must be included in the model to account for unmarked genetic effects. Knowledge of the fraction of the genetic variance not explained by markers is also required to predict the accuracy of the genomic predictions for each individual in the population, since it affects the maximum accuracy that can be achieved [17].

The base population correction of the genomic relationship matrix generally affected neither the proportion of genetic variance captured by markers, nor the genetic variance captured by the pedigree-based relationship matrices, which agrees with [17,30] but not with [31]. The latter authors, however, scaled the relationships in the opposite direction, i.e. when G relationships were too high, they scaled all relationships downwards, which further decreased the differences in relationships that were already small since relationships are bound by a maximum of 1 (and vice-versa when G relationships were too small). Moreover, the correction for the base population facilitates the integration of relationship matrices **A** and **G** into a single matrix (**H**), according to Legarra et al. [32], Christensen and Lund [13], and Meuwissen et al. [15].

We also estimated *C*_*miss*2_ using the pedigree-based estimate of genetic variance. The denominators of *C*_*miss*_ and *C*_*miss*2_ were significantly different from each other but both estimates revealed that the genomic relationship matrix could explain more than 95% of genetic variance if sufficiently reliable phenotypes are used (with reliabilities greater than 95%).

It should be noted that the estimates of *C*_*miss*_ and *C*_*miss*2_ depend on the SNP chip used, i.e. more dense SNP chips are expected to yield lower estimates of *C*_*miss*_ and *C*_*miss*2_ (a larger fraction of the variance is explained by the SNPs), and also on the family structure of the population [33]. Populations with more closely related individuals are expected to yield high LD between SNPs and QTL, even when they are physically quite far apart and, therefore, lower estimates of *C*_*miss*_. The population structure of the Italian Brown Swiss population reflects that of a typical dairy breeding population, and, thus, our results probably apply also to other dairy breeding populations.

## Conclusions

The fraction of genetic variance explained by genetic markers from high-density SNP panels was significantly different from 0 for the complex traits analyzed when the phenotypes are not highly accurate. The minimum fraction of the genetic variance not explained by the markers (*C*_*miss*_) was equal to 0.2, which was estimated based on the most accurate phenotypes. This value agrees with other values reported in the literature. Correction of the genomic relationship matrix for the variance of the allele frequency of each locus (**G**_**Y**_) instead of the average frequency of heterozygotes (**G**_**V**_), hardly explained any additional genetic variance. Our estimate of *C*_*miss*_ of 0.2 implies that about 80% of the genetic variance is explained by the Illumina 54 K SNP chip. Values for *C*_*miss*_ are expected to depend on the density of the chip (a larger SNP chip is expected to explain a larger fraction of the genetic variance) and on family relationships in the population, i.e. closer family relationships are expected to reduce *C*_*miss*_.

## Competing interests

The authors declare that they have no competing interests.

## Authors’ contributions

SIRP performed the study and drafted the manuscript. ABS contributed to writing the draft. SIRP, MAD and AB prepared the genotypic and phenotypic data. THEM planned and coordinated the whole study, and contributed to writing the manuscript. All the authors read and approved the final manuscript.
